# Ultrasound-guided percutaneous retrieval of non-radiopaque radial line using a microsnare

**DOI:** 10.1186/s42155-023-00407-5

**Published:** 2023-11-29

**Authors:** Hasan Alaeddin, Amr Elsaadany, Mohammad Rashid Akhtar

**Affiliations:** 1 Royal Derby Hospital, 14 Prothero Gardens, London, NW4 3SL UK; 2 Royal London Hospital, London, UK

**Keywords:** Radial artery, Retained catheter, Ultrasound, Minimally invasive techniques, Microsnare

## Abstract

Radial arterial lines are inserted in critically ill patients admitted to hospital intensive care units for continuous monitoring of their blood pressure. On removal the line can rarely become transected, potentially leading to thrombosis of the radial artery. Retrieval of the broken fragment can be achieved by open surgery, however other retrieval methods using ultrasound-guidance have been performed as they are considered safer and less invasive. We describe our technique of ultrasound-guided percutaneous retrieval of a broken non-radioopaque radial line in one patient, which involved the use of a microsnare. Under local anaesthesia and ultrasound guidance, a 6 Fr 5.5 cm short brite tip sheath was introduced into the radial artery, followed by a microsnare which was used to capture the arterial line, track the line back into the sheath and remove it uneventfully. The use of a microsnare under ultrasound-guidance is only one method to retrieve transected radial lines, with other interventional methods described in the literature. It enables a minimally invasive and safer approach to this potentially critical challenge and can help affected patients avoid open surgery to achieve the same management outcome.

**Supplementary Information:**

The online version contains supplementary material available at 10.1186/s42155-023-00407-5.

## Background

Radial artery catheterization, also commonly known as ‘radial arterial line’ insertion, is usually performed in critically ill patients who are admitted to intensive care units (ICU) for continuous real-time monitoring of their blood pressure and sampling of their arterial blood. Alternatively, they are also inserted in patients who are undergoing major procedures or surgeries [1]. As with any invasive procedure, complications can occur with radial artery catheterization (although it is generally viewed as a safe procedure). This procedure is predominantly performed by trained anaesthetists without ultrasound guidance.

Common complications include haematoma formation and temporary arterial occlusion [2]. A rare but serious complication is inadvertent transection of the catheter on its removal, which can cause thrombosis of the radial artery if not retrieved, and in patients who have an occluded ulnar artery or incomplete palmar arch it can lead to critical hand ischaemia [2]. There are a limited number of cases found in the medical literature detailing cases of retained radial artery catheter fragments caused by accidental catheter transection during removal [2–4].

Surgical removal of the retained catheter fragment is a relatively low-risk procedure but remains significantly invasive with questionable benefit in an otherwise asymptomatic patient. An alternative method of removal involves the use of ultrasound and local anaesthetic for an interventional procedure. The ultrasound-guided retrieval is minimally invasive, reducing the need for open surgery whilst also reducing patient discomfort and recovery time. The procedure is monitored in real-time, enhancing precision and minimising the risk of complications. In addition, ultrasound-guidance can be applied in various clinical settings, making it a versatile tool for broken radial line retrieval.

**Fig. 1:**
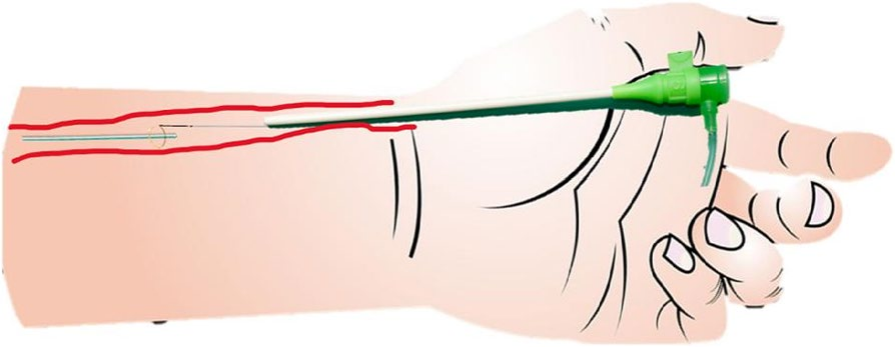
Illustration of percutaneous retrieval of retained radial line using microsnare technique

We describe the successful and uncomplicated ultrasound-guided percutaneous radial line fragment retrieval technique using a microsnare (Fig. 1).

## Case description

A patient was hospitalized in the intensive care unit at the Royal London Hospital after sustaining a renal injury from a road traffic accident that required embolisation. On admission they had a 20-gauge radial arterial line inserted in both arms for dynamic blood pressure monitoring. A few days later the patient was discharged from ICU and their arterial lines were all removed. However, after removal of the left radial line it was noted by the nursing staff that the proximal fragment of the line was missing. An ultrasound scan was then used to visualise the left radial artery and this demonstrated the missing catheter fragment along with some intimal defects/flaps within the radial artery which were not flow-limiting.

**Fig. 2:**
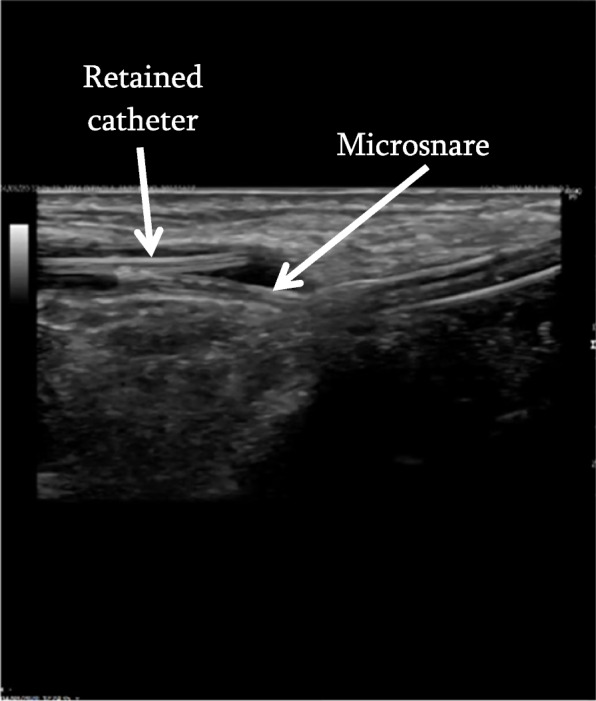
Ultrasound image demonstrating microsnare capturing the retained arterial line fragment

**Fig. 3:**
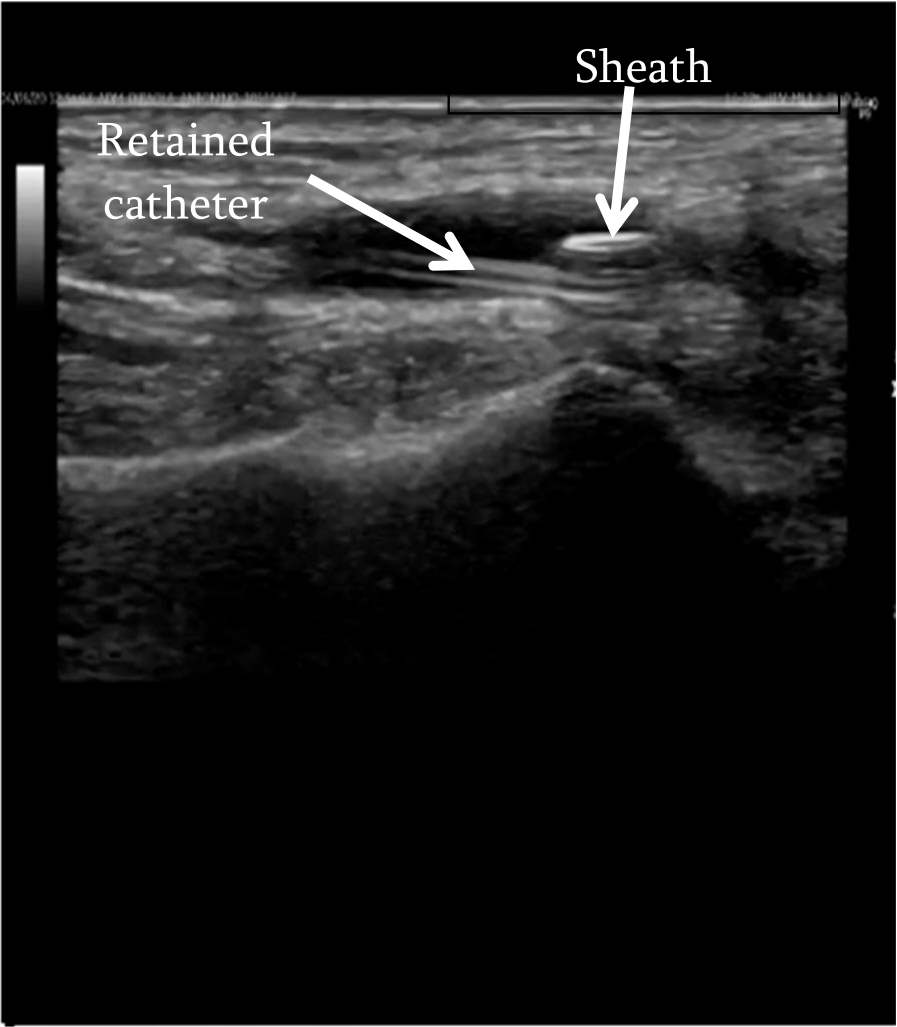
Ultrasound image demonstrating tracking back of retained fragment into sheath

**Fig. 4:**
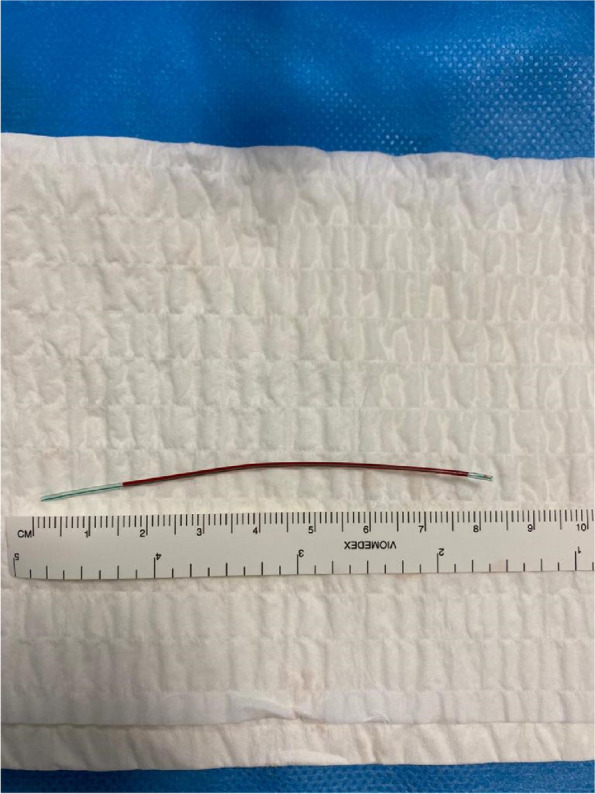
Retained catheter fragment post-removal

For the retrieval procedure, 1% lidocaine local anaesthetic was applied to the subcutaneous tissues of the left wrist, overlying the left radial artery above the radial styloid. A 4 French (Fr) micropuncture sheath (S-MAK, Merit medical, South Jordan, UT, USA) was inserted into the radial artery at this site under ultrasound guidance. The 4 Fr micropuncture sheath was exchanged under ultrasound guidance for a 6 Fr 5.5 cm short brite tip sheath (Cordis, Miami Lakes, FL, USA). This was followed by the insertion of a microsnare (3 Fr Amplatz Goose Neck, ev3 Inc., MN, USA) into the sheath. Under continuous ultrasound visualisation, the opened microsnare was used to capture the arterial line fragment (Fig. 2) (video 1). The fragment was then tracked back into the sheath and removed (Fig. 3) (video 2). After confirmation of removal of the entire fragment, the 6 Fr sheath was removed and a transradial band (TR Band, Terumo, Tokyo, Japan) was then applied to the puncture site to help stem bleeding and assist haemostasis. There were no complications reported post-procedure (Fig. 4).

## Discussion

This case involved the use of a microsnare under ultrasound-guidance to retrieve a radial arterial line fragment that had been transected during line removal. This percutaneous approach enabled the minimally invasive retrieval of the line fragment without the need for open surgery. Such an approach requires high-quality ultrasound imaging and familiarity with ultrasound-guided endovascular procedures. To our knowledge, this is the first case in the literature detailing the use of a microsnare in such a technique for retrieval of a radial artery catheter fragment under ultrasound guidance. A previous case report in the literature describes the ultrasound-guided retrieval of broken radial lines in two patients, with one case using a Fogarty arterial embolectomy catheter to retrieve the line, and in the second the retrieval was achieved by cannulating the fragment with a non-hydrophilic catheter [5].

## Conclusion

Ultrasound-guided retrieval of broken radial lines can be a valuable technique in interventional radiology and vascular procedures. The use of a microsnare under ultrasound-guidance offers a minimally invasive, precise, and versatile approach to address this potentially critical medical challenge, and can help avoid the need for open surgical procedures to achieve the same management outcome for affected patients.

### Supplementary Information

**Additional file 1.**

**Additional file 2.**

## Data Availability

Not applicable.

## Abbreviations

ICU: Intensive care unit
Fr: French
