# Correction: Ultrasound-guided percutaneous retrieval of non-radiopaque radial line using a microsnare

**DOI:** 10.1186/s42155-023-00419-1

**Published:** 2024-01-03

**Authors:** Hasan Alaeddin, Amr Elsaadany, Mohammad Rashid Akhtar

**Affiliations:** 1 Royal Derby Hospital, 14 Prothero Gardens, London, NW4 3SL UK; 2 Royal London Hospital, London, UK

Correction: *CVIR Endovasc ***6**, 59 (2023)

10.1186/s42155-023-00407-5.

Following the publication of the original article [1], the authors would like to update the corresponding author’s affiliation to “Royal Derby Hospital, Derby, UK.”.
